# Mass flow and momentum flux in nanoporous membranes in the transitional flow region†

**DOI:** 10.1039/d1cp02797b

**Published:** 2021-07-26

**Authors:** Stepan K. Podgolin, Dmitrii I. Petukhov, Thomas Loimer, Andrei A. Eliseev

**Affiliations:** Department of Materials Science, Lomonosov Moscow State University 1-73 Leninskiye Gory Moscow 11 9991 Russia di.petukhov@gmail.com; Department of Chemistry, Lomonosov Moscow State University 1-3 Leninskiye Gory Moscow 11 9991 Russia; Institute of Fluid Mechanics and Heat Transfer, TU Wien 1060 Vienna Austria

## Abstract

An experimental study of momentum transfer in nanoporous polymeric track-etched membranes with pore diameters ranging from 100 to 1300 nm and nanochannel lengths of 12–20 μm was performed using He, N_2_, CO_2_, and SF_6_ propellants in a wide range of plenum and background pressures. Mass flux through the membranes was elaborated as a combination of Knudsen diffusion and viscous flow at Knudsen numbers above 0.1 and become choked at lower Knudsen numbers. The discharge coefficient for the membranes attained was 0.6, making the permeation rate similar to that of thin orifices. The effect is attributed to the mirror reflection of the molecules from the pore walls at low angles of incidence. The exhaust gas velocity is found to be dependent on the plenum to background pressure ratio and channel length-to-diameter ratio, reaching 0.9 of the velocity of the gas expanded to vacuum (up to 2 M). Close to an isothermal expansion occurs in nanochannels of all sizes. A general quantitative description for gas expansion in nanochannels is provided. The highest thrust is generated in the choked flow regime with the SF_6_ propellant and a value of 4.5 N cm^−2^ is attained at a propellant consumption of 0.165 kg (cm^2^ s)^−1^ for the membranes with 1300 nm nanochannels. The specific impulse of 138 s is reached for helium. The results show the prospects of the utilization of nanoporous membranes in cold gas propulsion systems.

## Abstract 2

An experimental study of momentum transfer in nanoporous polymeric track-etched membranes was performed using He, N_2_, CO_2_, and SF_6_ propellants. The exhaust gas velocity reaches values of up to 2 M. Close to an isothermal expansion occurs in the nanochannels.

## Graphical abstract

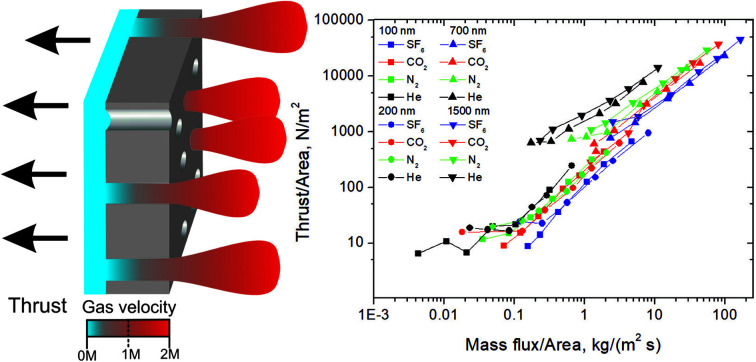

## 1. Introduction

Interactions of penetrant molecules with permeable media are undeservedly paid little attention in modern sciences. In microfluidics and propulsion systems, the nozzles and orifices are usually considered neglecting the interactions of propellants with the orifice walls, while in membrane sciences the penetrants aren’t supposed to transfer impulse to membranes.^1–3^ Meanwhile, in both these areas, the trend of continuously reducing the channel sizes to sizes comparable with molecule jump lengths or mean free traveling paths suggests an increasing role of particle-wall interactions. On the other hand, these interactions can introduce a significant effect into a general gas expansion law. Indeed any molecule–wall collision can be regarded as equating the temperature of a gas with the local temperature of the walls. This becomes especially important in the transitional flow region when the number of gas–gas and gas–wall collisions become of the same order. The walls, coincidently, can provide a high enough heat conductivity with the necessary thermal input for the propellant flux to achieve close to an isothermal expansion and affect both the momentum and heat transfer efficiency.

We consider the use of nanochannels instead of orifices, which will enable regulation of heat transfer to the permeating medium, allowing control over the gas expansion process. With this, a number of important issues appear including the possibility of effective thrust generation, cooling and heat extraction from low-grade heat sources. Moreover nanochannel gas nozzles allow independent study of both the effects of the plenum and background pressures on the momentum transfer efficiency and provide the option of integrating heaters or accelerating electrodes for heat or electrically powered propulsion systems.^4,5^ They also hold promise for the easy design of cold gas micro-thrusters with a μN–mN range for micro- and nanosatellites.^6^

At the same time, reducing the channel size and operation at low pressures leads to a transition from a continuous gas flow regime to free-molecular flow. Traditionally the gas transport regime is described using the Knudsen number, which is defined as the ratio of gas molecule mean free path (*λ*) to a characteristic dimension of the channel (*d*).^7^ In nanoporous channels with a pore diameter in the order of 100–1000 nm operating in a pressure range from several mbars to about 1 bar a typical Knudsen number varies from 0.01 to 30, corresponding to the transitional and free molecular flow.

At low Knudsen numbers (<0.01), the gas flow can be described by the Navier–Stokes equation applicable for a continuous medium and defined mostly by the pressure gradient. At high Knudsen numbers, the free molecular flow becomes the predominant transport mechanism with no momentum transfer through intermolecular collisions and an average gas molecular velocity equal to the thermal velocity. However, only a few studies report on the investigation of momentum flux at various Knudsen numbers. Ziarani *et al.*^8^ and Alexeenko *et al.*^9^ performed experimental and numerical analyses of low thrust propulsion systems based on circular orifices with a diameter of 1 mm in the range of Knudsen numbers from 0.005 to 40 using helium, nitrogen, and argon propellants, providing thrust values ranging from 0.14 to 3 mN. Approximation of experimental data using a continuum flow shows perfect agreement for Knudsen numbers below 0.01, while no approximations were given for high Knudsen numbers. Lilly *et al.*^10^ performed a comparison of thrust generated by orifices with a diameter of 1 mm and various *L*/*D* ratios ranging from 0.5 to 1.2 in the same experimental conditions and demonstrated that the thick orifice propulsion efficiency is much higher than that for thin orifices. However, to the best of our knowledge, the experimental results for nanometer scale channels and orifices have never been exposed. No impulse transfer studies were performed for nanochannels in the free-molecular flow regime. The lack of data is most probably connected with too low flux and thrust values provided by individual nanochannels.

Meanwhile recent progress in micro- and nanofabrication enables the preparation of nanochannel membranes with controlled diameters and pore density, narrow pore size distribution, and reproducible porous structures, such as anodic alumina^11,12^ or track-etched membranes.^13^ The uniformity of the channels in those systems allows them to be considered as identical nanochannel arrays performing as individual elements. Despite the high breaking strength of anodic alumina^14^ its ceramic nature leads to high brittleness of the membranes and a limited ability to operate under rapid pressure changes. Thus, here we focused on the experimental elaboration of impulse transfer in the transitional flow region with nanoporous track-etched membranes with nanochannel sizes of 100–1500 nm and a thickness to diameter ratio varied in the range from 200 to 8. Several gases including helium, nitrogen, carbon dioxide, and sulfur hexafluoride with molecular weights from 4 g mol^−1^ to 146 g mol^−1^ were utilized as propellants at inlet pressures of 0.5–4 bar and exhaust pressures of 0.01–0.5 bar, providing average Knudsen numbers of 9.8 × 10^−3^ to 3.16. To the best of our knowledge, this is the first work exhibiting experimental data on impulse transfer in nanochannels in transitional and free molecular flow regimes.

## 2. Experimental

Polymeric track-etched (TE) membranes with nominal pore diameters were purchased from Trackpore Technology CJSC (Russia) and were used as received. The average pore diameter, pore size distribution, and porosity of samples were examined using a Leo Supra 50VP (Carl Zeiss) scanning electron microscope with subsequent statistical analysis of the images using ImageJ software.

**Fig. 1:**
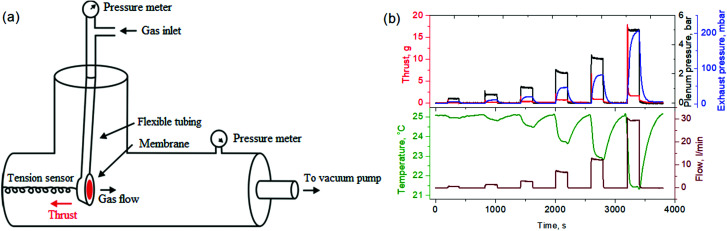
(a) Scheme of the experimental setup and (b) a typical dataset acquired in a gas transport/thrust measurement experiment.

The experimental setup for registering gas flow parameters and momentum transfer in the nanoporous media comprised a membrane cell suspended on flexible tubing and resting on the tension sensor installed in the vacuum chamber (Fig. 1a). Two preliminary calibrated tension sensors (XIN NUO QI) with upper limits of 20 g and 100 g were utilized. The vacuum chamber with a 32.15 L total internal volume was evacuated with an ERSTEVAK ESVP 150 vacuum pump (pump speed 2 L s^−1^). The gas flow and plenum pressure were regulated with a needle valve. Gas flow through the membrane during the experiment was measured using a Brooks SLA5850 mass flow meter (Brooks, USA). The plenum and background pressures were recorded using Carel SPKT (USA) pressure transducers installed directly before the membrane cell and a high-accuracy Piezus (Russia) pressure transducer located in the vacuum chamber. The permeate temperature was registered with a Type K thermocouple installed downstream of the membrane cell at a distance of 2 mm. The propellant was supplied from a gas cylinder installed outside the vacuum chamber. He, N_2_, CO_2_ and SF_6_ were utilized as propellants.

In a typical experiment, an approximately constant mass flow rate, set with a needle valve, and calibrated with a mass flow meter was supplied to the preliminary evacuated membrane cell by the instant opening of the ball valve connected in series. In this case, the filling time for reaching steady-state plenum pressure didn’t exceed 30 ms. The ball valve was kept open until stabilization of thrust and background pressure (∼100 seconds), see Fig. 1b. Typically, the experiment was repeated 3 times at each flow/plenum pressure condition. After each cycle, the system was fully evacuated (∼100 seconds). Continuous monitoring of thrust, mass flow, pressure and temperature during the experiments was carried out using a self-developed hardware/software system with a recording interval of 100 ms.

## 3. Results and discussion

**Fig. 2:**
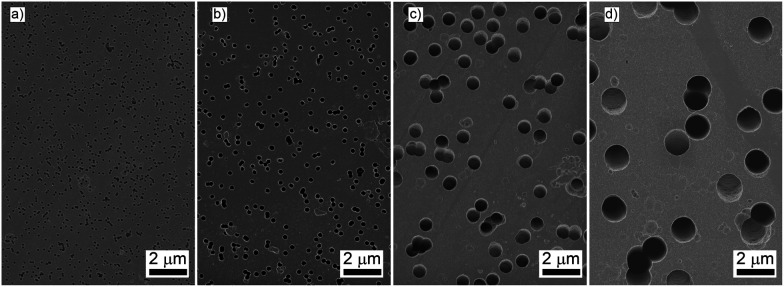
SEM images of track-etched membrane with different pore diameters: TE_100 nm (a), TE_200 nm (b), TE_700 nm (c), TE_1500 nm (d).

Prior to gas transport experiments the porous structure of polymeric track-etched (TE) membranes with different pore diameters was thoroughly analysed with a scanning electron microscope in order to determine the average pore diameter, pore size distribution, and porosity (Fig. 2). The results of the statistical analysis of the SEM images are listed in Table 1. Corresponding pore size distribution values extracted using statistical analysis of the SEM images are provided in the ESI 1 (ESI†). The half-width of the pore size distribution does not exceed 5% as was previously reported for track-etched membranes.^13,15^ With the same pore diameters found on the two sides of the membranes, and well-defined anisotropy of the tracks we assumed a cylindrical shape of the pores. Thus, all further calculations were provided accounting for the cylindrical pore model.

**Table 1:** Characteristics of utilized porous systems

Membrane	Membrane area, cm^2^	Nominal pore diameter, nm	Porosity, %		Thickness, μm	*J*_Kn_√*M*, m^3^(STP) kg^0.5^ m^−2^ bar^−1^ h^−1^ mol^−0.5^	Maximum thrust, N m^−2^	Maximum speed, M	Maximum specific impulse, s
			From SEM	From gas permeance measurement					
TE_100 nm	1.78	120	9.4	8.0	20	41.93	650	0.93	40
TE_200 nm	1.78	200	7.6	8	17.5	79.87	931	0.76	37
TE_700 nm	0.096	630	9.1	10.5	12	458.6	22300	1.56	116
TE_1500 nm	0.0259	1300	12.3	13	12	1200.3	34500	1.79	138

The porosity parameters of the nanochannel membranes were utilized for calculating Knudsen permeance and further comparison with the experimental values using the conventional relation:1
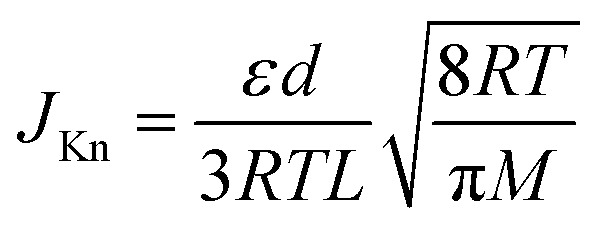
where *J*_Kn_ – permeance in a Knudsen flow regime, *ε* – flow-through porosity, *d* – average pore diameter, *L* – membrane thickness, *M* – molecular weight of the penetrating gas, the pore tortuosity was taken to be equal to one. Notably, eqn (1) contains the molecular weight of the penetrant, while 
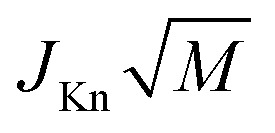
 can be regarded as a permeate-independent membrane characteristic. Thus this parameter was further utilized in the considerations (Table 1).

**Fig. 3:**
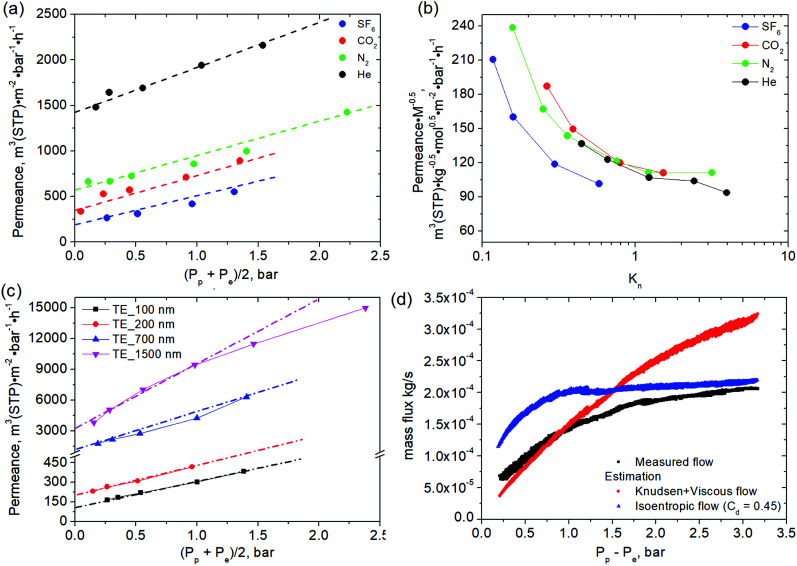
The dependence of gas permeance for the TE_200 nm membrane *vs.* mean pressure (a) and average Knudsen number (b). The permeance of track-etched membranes with various pore diameters for SF_6_. The linear regressions in (a) and (c) are given assuming viscous flow contribution according to eqn (2). (d) The dependence of mass flux through the membrane with 1500 nm pore diameter *vs.* pressure difference at a fixed plenum pressure of 3 bar and varied background pressure, the estimated values of mass flux assuming realization of transitional flow regime (combination of viscous and Knudsen flow) and isoentropic flow with a discharge coefficient equal to 0.45 are added as references.

Experimental determination of 
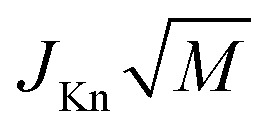
 consisted of measuring stationary gas fluxes at variable plenum and background pressures. The derived dependences reveal close to a linear growth of membrane permeance with average pressure (half-sum of the plenum and background pressures), indicating the viscous flow contribution (Fig. 3a). According to the dusty gas model, the contributions of viscous and diffusive (Knudsen) fluxes are additive^16^ and the Knudsen contribution does not depend on average pressure.^17^ Thus, Knudsen permeances for individual gases can be extracted by interpolating the pressure dependence on the ordinate axis (at (*P*_p_*+ P*_e_)/2 = 0). With this model, gas-normalized permeances, 
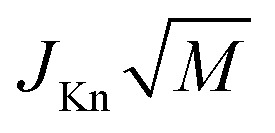
, for each membrane consolidate into a unified dependence on the averaged Knudsen number (Fig. 2b), and are acceptably fitted by the equation suggested in ref. 17:2
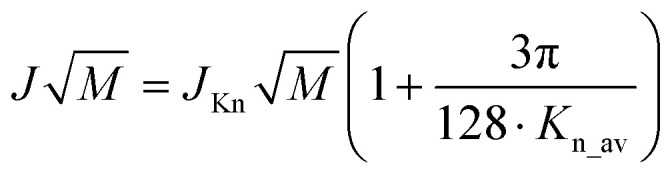
The first term in eqn (2) corresponds to the Knudsen contribution and the second to viscous flow. *K*_n_av_ – represents an average Knudsen number calculated with plenum (*P*_p_) and background (*P*_e_) pressures by:3
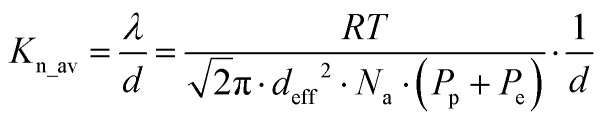
with *N*_a_ – Avogadro number, *d*_eff._ – gas molecule collision diameter.

**Fig. 4:**
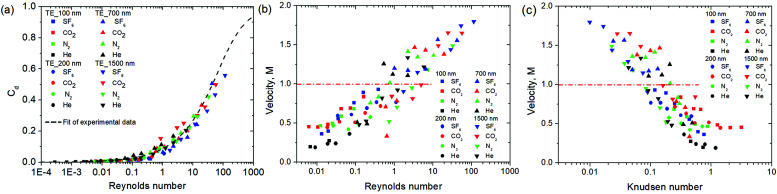
Dependence of discharge coefficient on Reynolds (a) number calculated based on the ratio of experimental mass flux to choked flow. The fitting of experimental data is represented by a dashed line. The dependencies of exhaust gas velocity normalized to velocity of sound on Reynolds (b) and Knudsen (c) numbers.

However, theoretical permeances deviate from the experimental values, especially for heavy gases permeating large pores. The deviation generally increases with plenum pressure (Fig. 3b). The difference can partly be explained with *K*_n_ variation along the channels and the contribution of intermolecular collisions.^17^ However, significant flux limitation at low *P*_out_ unambiguously points to flow choking (Fig. 3d), with the mass flow rate limited solely by plenum conditions. The estimate for the choked flow with a discharge coefficient of *C*_d_ = 0.45 illustrates a perfect approximation for flux limitation with:4
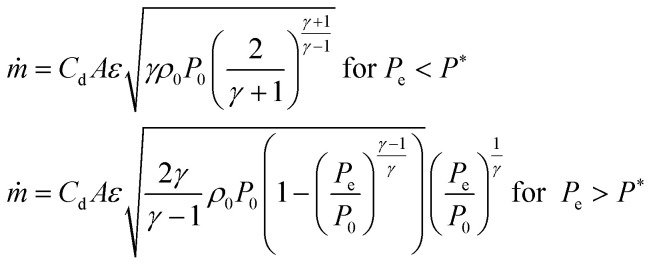
where *A* – membrane area, *ε* – membrane porosity, *γ* – heat capacity ratio of gas, *P*_0_ – plenum pressure, *ρ*_0_ – gas density at plenum pressure and temperature. The deviation of experimental results from the Knudsen + viscous model (at low pressures) can be caused by the contribution of slip flow, attributable to specular reflections of gas molecules from the walls.^17^ We also can’t exclude the role of larger (overlapped) pores in excessive flow contribution.^18,19^ For higher pressures, hence higher mass flow rates and higher Reynolds numbers, the difference can also be caused by the increased flow resistance in the entry region. In the entry region, the friction factor is larger than for fully developed flow.^20^ To further analyse the apparent limitation, we estimated the discharge coefficients for the experimental fluxes using eqn (4). The dependence of discharge coefficient *vs.* Reynolds numbers indicates a perfect relation, independent of *L*/*D* ratio, which varies two orders of magnitude in the experiments (Fig. 4a, see also the same dependence in the Log–Lin coordinates in the ESI 2, ESI†). The best fit equation,5
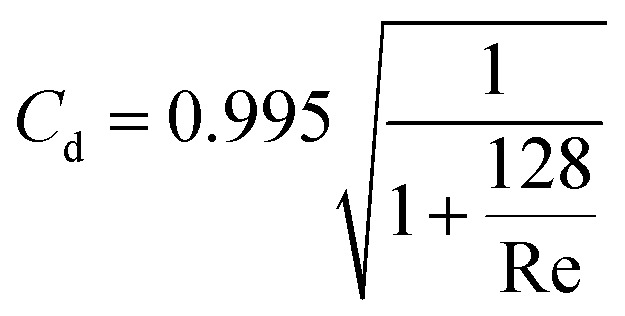
coincides rather well with earlier published data for large tubes.^21^ Surprisingly, the discharge coefficients attaining values of 0.6 were resolved with the calculations. This makes the areal flux through the nanopores similar to that of macro-scale holes or even thin orifices, which is unexpected from intuitive knowledge. This seems most surprising accounting for large *L*/*D* ratios and the estimated number of collisions suffered by the molecules in the pores. Those vary from 10 for the membranes with the largest pore diameter to ∼1000 in 100 nm pores. Despite the general tendency of increasing *C*_d_ with Reynolds number being well known for macro-scale ducts, high absolute *C*_d_ values and flow choking suggest sonic flow velocities are attained in the nanochannels, which requires further analysis.

To resolve the gas flow regime in the nanochannels, impulse transfer investigations were performed by measuring the thrust generated by gases passing through the nanoporous membranes. Both plenum and exhaust pressures were varied independently during the measurements. That was realized with a slow filling of the large permeate side volume with gas, permeating through the membranes under varied plenum side pressures. The plenum and exhaust pressures, propellant consumption, and output temperature were continuously monitored during the experiments at 0.1 s intervals. A typical acquired dataset is given in the original timescale in Fig. 1b. With the step-like increase of plenum pressure, thrust quickly reaches a maximum value, which then diminishes with pressure growth in the exhaust volume until reaching stationary conditions (typically in 50-100 s). An apparent exhaust gas velocity (*ν*_e_) can be defined using the equation for momentum flux, which is calculated from the generated thrust (*F*, see ESI 3, ESI†) and mass flow rate (*ṁ*):6
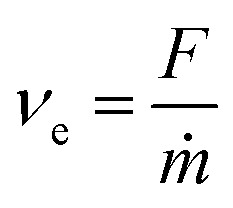
To unify the data for various gases, exhaust gas velocities were further normalized to the velocity of sound at the upstream condition:7
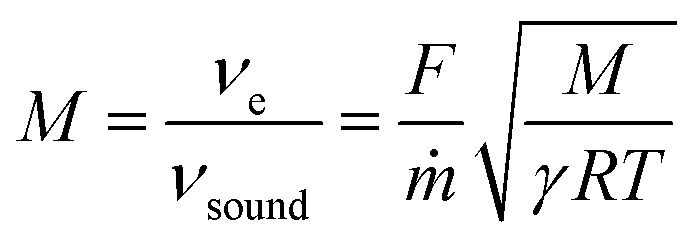
Exhaust gas velocities, extracted from impulse transfer for different membranes and propellants, were plotted *vs.* Reynolds (Fig. 4b) and Knudsen numbers (Fig. 4c). These reflect the differences and similarities of the flow conditions in the nanochannels of various sizes. Impulse transfer from gases, permeating through the membranes with low pore diameter (*D*_pore_ = 100–200 nm) in the molecular flow regime (high *K*_n_ and low Re) corresponds well to 1/3 of the thermal velocity and is in accordance with theoretical predictions. With decreasing *K*_n_, the contribution of intermolecular collisions increases, accelerating gas molecules to an average velocity approaching *M* = 1 at *K*_n_ ∼ 0.1 (Re = 1). The effect becomes more pronounced for large diameter pores (*D*_pore_ = 700–1500 nm), crossing the 1M line. Further densification of fluid results in supersonic impulse recovery attaining a calculated velocity of 2 M in certain cases.

However, choked flow in a straight duct is usually considered to be limited by flow velocity up to 1 M, excluding further gas expansion. The limit of 1 M is independent on the energy influx or friction and corresponds to the maximum entropy state. Thus, impulse recovery from the supersonic flow needs further explanation. The result looks most surprising, considering the velocity evaluation method that measures membrane cell thrust, which suggests interpretation of the derived value as an exit velocity of the medium. On the other hand, an estimation of the maximum achievable exhaust gas velocity, accounting for the final and initial states for a compressible gas flowing through an orifice with a ratio of plenum to background pressure of about 100, results in ∼2.5 M.^22^

The attribution of recovered impulse and flux through the nanochannels requires the transport mechanism and pressure profiles in the nanochannels to be resolved. Two possible models can be drawn here. The first involves subsonic acceleration in the nanochannels with further gas expansion after the exit plane. The second presumes possible acceleration to supersonic velocity before the pore exit, similar to the model derived for a straight duct with modeling.^23^ The approaches differ in gas pressure and velocity at the exit plane. For the first case, the total force affecting the membrane can be expressed as the sum of in-channel flow acceleration and volume gas expansion behind the membrane:

8 *F* = *ṁv*_e_ + *εA*(P* − *P*_e_)

Despite the second term in eqn (8) not mechanistically explaining the impulse transfer to the membrane, it resolves the problem of supersonic exhaust velocities formally. However, it fails to explain very small temperature variations of the exhaust gases (Fig. 1b). Indeed, with energy conservation, adiabatic expansion of a supersonic gas with a gain of kinetic energy should result in a strong decrease of its temperature, which contradicts the experimental data. The temperature sensor installed exactly in front of the membrane cell at a distance of ∼2 mm indicates no significant cooling even at large flow rates (of 10 l min^−1^) and with heavy gases. Generally, experimental temperature variation doesn’t exceed the Joule–Thompson effect. Accounting for the very similar geometry of our experiment to the one of Joule–Thomson, we can attribute the temperature variations to gas expansion after the membrane exit. This implies close to isothermal conditions for the gas until the pore exit.

**Fig. 5:**
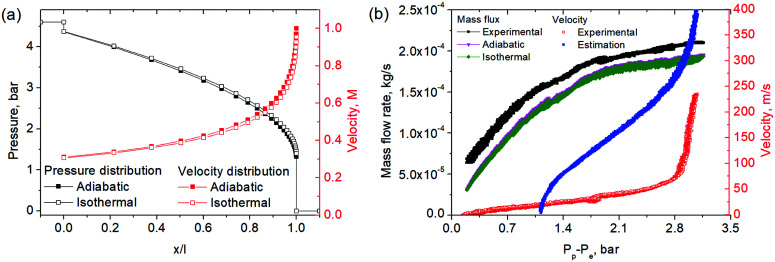
(a) Pressure and velocity distribution along a nanopore for SF_6_ flow through the membrane with *D*_pore_ = 1500 nm, *P*_in_ = 4.66 bar, *P*_out_ = 0.0038 bar (b) the dependence of mass flux and exhaust gas velocity *vs.* transmembrane pressure.

To determine the nature of the gas expansion process in nanochannels, we performed a theoretical calculation of the mass flux and attainable velocities for the cases of compressible isentropic, adiabatic and isothermal flows using equations suggested in ref. 24 as well as the combination of molecular and viscous flows^17^ (the calculation scheme is given in the ESI 4, ESI†). The description of continuum flows involved calculation of the pressure and velocity profiles, necessary for determining exit plane conditions (Fig. 5a). The comparison of theoretical and experimental results (Table 2 and Fig. 5b) illustrates a rather good agreement with the isothermal model for the description of experimental data for mass flux. At the same, time the expected velocity at the exit plane exceeds the sound velocity, indicative of in-pore supersonic expansion for adiabatic and isothermal flow.

**Table 2:** A comparison of different gas expansion mechanisms for SF_6_ flow through a membrane with *D*_pore_ = 1500 nm *P*_in_ = 4.66 bar, *P*_out_ = 0.0038 bar (sound velocity is equal to 136 m s^−1^)

	Mass flux, kg s^−1^	Critical pressure, bar	Velocity on the exit plane, m s^−1^
Experiment	4.28 × 10^−4^	—	270
Knudsen + Viscous	6.55 × 10^−4^	—	69
Adiabatic	3.55 × 10^−4^	1.35	170
Isentropic	7.16 × 10^−4^	2.73	68
Isothermal	3.51 × 10^−4^	1.43	164

**Fig. 6:**
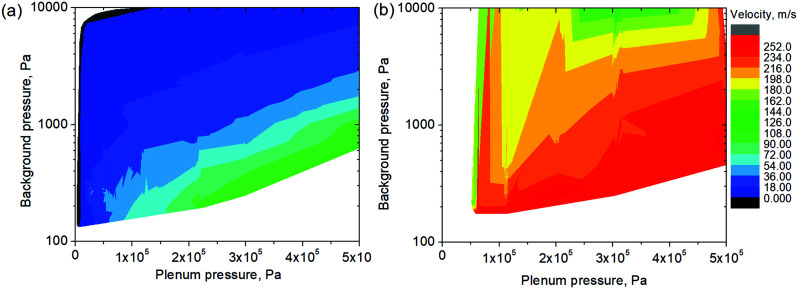
The dependence of exhaust SF_6_ velocity on plenum and exhaust pressures for nanochannels operating in a Knudsen/viscous flow regime (TE 200 nm) and a choked flow regime (TE 1500 nm).

To resolve whenever the sonic line is crossed within the pore volume or outside the membrane, we analysed the role of pressure conditions for the enhancement of impulse transfer carefully (Fig. 5b). An intercomparison of flux and velocity dependencies on Δ*P* revealed only a weak correlation of flow choking (∼1.5 bar) and velocity upraise (0.2 bar) pressures. In the numerous experiments, the last always had a threshold of ∼20 kPa, revealing the role of exhaust pressure (Fig. 6). Generally, the exhaust velocities illustrate a strong dependence on *P*_p_/*P*_e_ ratio. This dependence becomes less pronounced on *P*_p_ for large channels (*D*_pore_ = 700–1500 nm). This kind of relation on plenum-to-exhaust pressure ratio is known for compressible flow through thin orifices in un-choked conditions.^24^ A similar relation can be extracted for viscous flow. It suggests a behaviour of the system similar to that of an orifice plate positioned at the exit plane of the nanochannel and operating in un-choked conditions. Notably, the pressure profiles calculated for the isothermal model also revealed a pressure drop at the end of the nanochannel.

With these results, in analogy with Dushman's relation,^25^ we propose consideration of the nanochannel as a long duct, with a choking pressure of some critical value and an orifice of the same diameter connected in series. As soon as the flow resistance of the orifice in most cases can be neglected, compared to a duct of the same diameter, the flux through the nanochannels can be determined in a conventional way with eqn (4). In the case of applying adiabatic or isothermal conditions (Fanno or Rayleigh flows) the corresponding relations can also be utilized (see ESI 4, ESI†). The impulse transfer and velocity of exhaust gases can then be calculated assuming orifice expansion of the gas, entering at sound velocity and a critical downstream pressure of the duct. This approach coincides principally with experimental data in the inflection points and provides rather a good approximation at low *P*_e_ (Fig. 6). Further refinement can determine the efficiency of impulse recovery.

The comparison of expansion in the nanochannels with conventional cold gas propulsion systems based on orifices clearly evidences a preference for the isothermal process. The maximum thrust generated at the choked flow conditions for propellants with high molecular weight attained is 4.5 N cm^−2^ at a propellant consumption of 1.65 × 10^−2^ kg (cm^2^ s)^−1^ for an SF_6_ and TE 1500 nm membrane. For plenum pressures exceeding 1 bar and background pressures in the range of 2 to 150 mbar, the resulting thrust to propellant consumption ratio of 270–300 m s^−1^ (1.8–2 M) is significantly higher than the values obtained for orifices by Lilly^10^ and Alexeenko^8^ using nitrogen or helium propellants. At the same time, the low condensation pressure of condensable SF_6_ and CO_2_ gases allows their storage in the liquid/solid state, providing an additional preference for cold gas propulsion system design. Using helium as the propellant, the specific impulse of 126 s is attained with nanochannel membranes (Table 1). The results show the prospects of using nanochannel media for further development cold gas propulsion systems with low propellant consumption for micro- and nanosatellites. We also believe that the presented results will contribute a fundamental understanding of flow mechanisms and impulse transfer in nanochannels and provide a background for creating novel flow accelerators for effective cooling, thrust generation and heat extraction.

## Conclusions

In summary the experimental study of momentum transfer in nanoporous polymeric track-etched membranes with pore diameters ranging from 100 to 1300 nm with He, N_2_, CO_2_, and SF_6_ propellants in a wide range of plenum and background pressures revealed the possibility of the isothermal expansion of gases with high attainable discharge coefficients and supersonic exhaust velocities at Re > 1. The model description of the expansion process and impulse transfer indicated the key role of plenum and background pressures and gas type in attaining maximum gained momentum. When reaching discharge coefficients close to unity and apparent exhaust velocities up to 2 M at the stagnation temperature, a number of important issues appear for gas expansion and transport in nanochannels with *L*/*D* ≫ 1 in the free-molecular flow and choked flow regimes:

(1) The nanopores can be utilized to achieve supersonic expansion with a lowering of background pressures. The pressure at the exit plane is considered the main parameter affecting the momentum flux. A model of one-dimensional flow provides underestimated impulse recovery, suggesting lower pressure at the exit plane of the nanochannels, compared to experimental data. The mass flux through long nanopores and transferred momentum can be estimated assuming flow through a straight duct and an orifice connected in series.

(2) A supersonic transition can occur within the membrane volume allowing further exploiting the properties of supersonic flows directly in nanochannel (*i.e.*, ionization and electric acceleration for nanobeams). Nanoporous membranes enable easy arrangement of μN–mN propulsion systems and manipulation of gas expansion levels with nanochannel diameter and the permeability of nanoporous membranes. Moreover, introducing the third dimension allows further design of electrode systems for linear acceleration and similar devices.

(3) The nanopores allow efficient heat transfer through the pore walls allowing isothermal expansion and enabling the use of electric heaters for thrust generation or efficient cooling.

We believe these exciting results will open numerous opportunities for novel realizations of gas propulsion and thermodynamic cycles and will serve as a baseline in supersonic nanofluidics.

## Conflicts of interest

There are no conflicts to declare.

## Supplementary Material

CP-023-D1CP02797B-s001
