# Supplementary material for: Mass flow and momentum flux in nanoporous membranes in the transitional flow region
Source: Phys Chem Chem Phys. 2021 Jul 26;23(32):17134–41. doi: 10.1039/d1cp02797b (PMC8489186; doi:10.1039/d1cp02797b)
Supplement: CP-023-D1CP02797B-s001 [file CP-023-D1CP02797B-s001.pdf]

Electronic Supplementary Material (ESI) for Physical Chemistry Chemical Physics.
This journal is © the Owner Societies 2021

Electronic supplementary information

Mass flow and momentum transfer in nanoporous membranes in the transitional flow region

Stepan K. Podgolin[a], Dmitrii I. Petukhov[a,b,*], Thomas. Loimer[c], Andrei A. Eliseev[a,*]

[a] – Department of Materials Science, Lomonosov Moscow State University, 1-73 Leninskiye gory, Moscow, 11 9991, Russia

[b] – Department of Chemistry, Lomonosov Moscow State University, 1-3 Leninskiye gory, Moscow, 11 9991, Russia

[c] – Institute of Fluid Mechanics and Heat Transfer, TU Wien, 1060 Vienna, Austria

Corresponding authors:

* - D.I. Petukhov di.petukhov@gmail.com

* - A.A. Eliseev eliseev@inorg.chem.msu.ru

SI 1. Pore size distribution of track etched membranes.

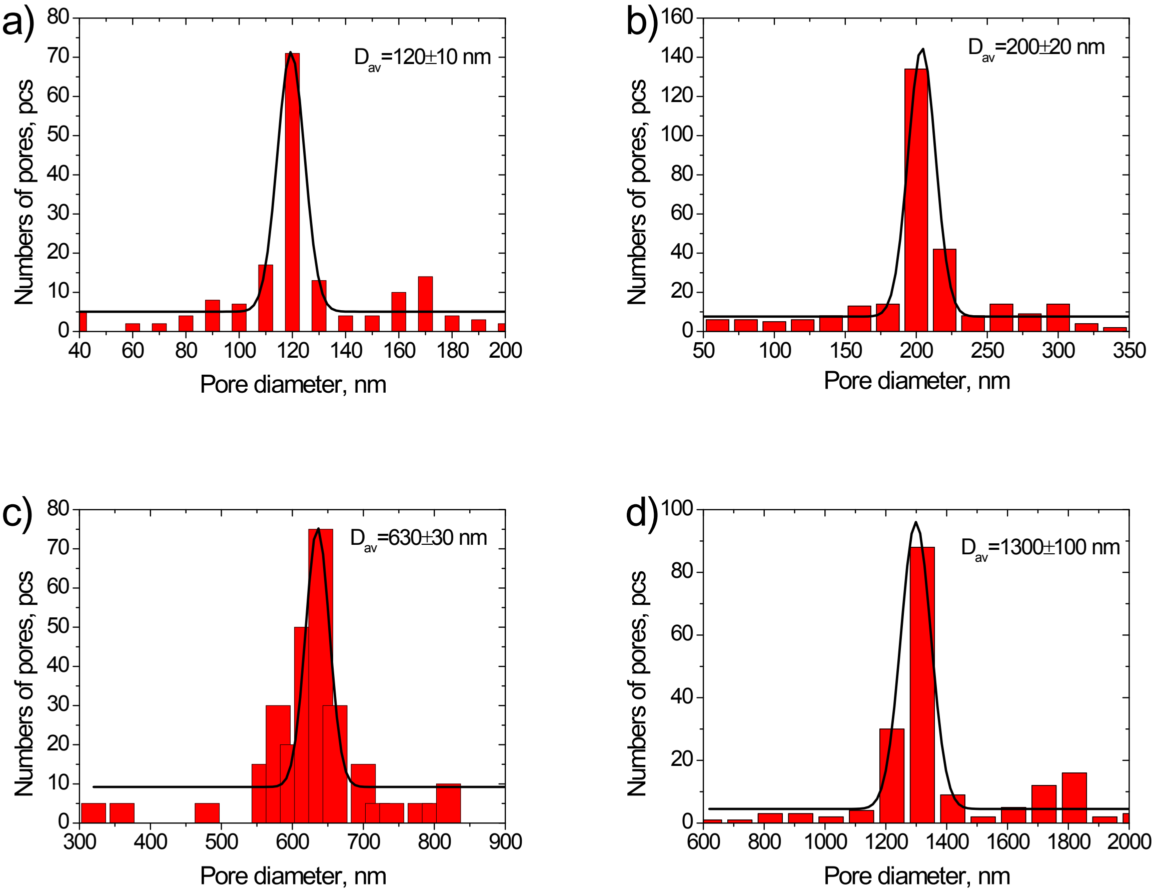

Figure SI1 – Pore size distribution obtained from SEM images of different track-etched membrane TE_100 nm (a), TE_200 nm (b), TE_700 nm (c), TE_1500 nm (d).

SI 2. Dependence of discharge coefficient vs Reynolds number

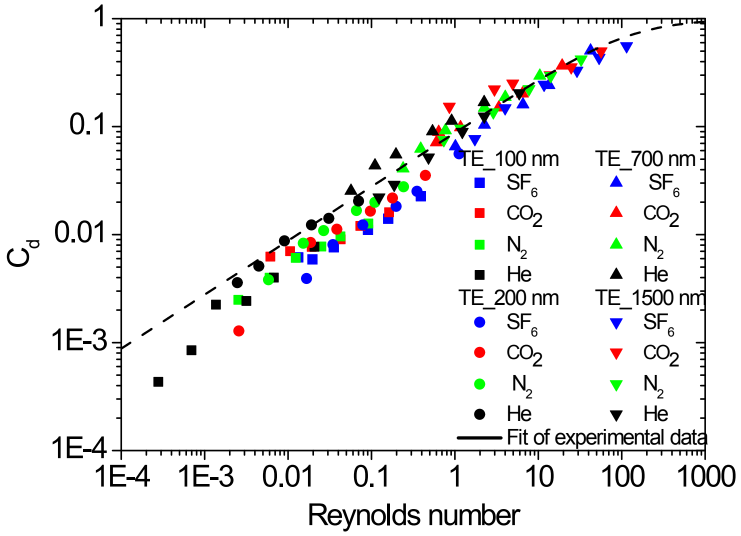

SI 3. The dependence of normalized thrust vs. normalized mass flow for helium, nitrogen, carbon dioxide and sulfur hexafluoride measured for all membranes.

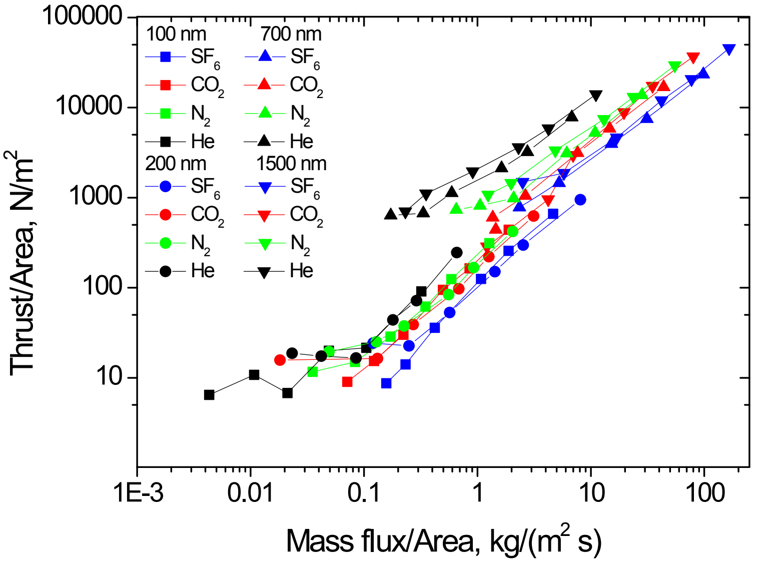

SI 4.

For expansion of a gas from approximately atmospheric conditions to near vacuum, the fluid velocity might reach the speed of sound and choked flow conditions may occur. In the foregoing, e.g., by application of Eq. (2), no account was taken of the possibility of choked flow. Hence, in the following, the flow is investigated by considering the purely viscous flow of a continuum through the pores. A rather crude model is applied, viz., it is assumed that the gas expands isentropically from its stagnation conditions, given by the plenum pressure and temperature, to the state at the upstream front of the membranes. For the flow through the membrane pores a fully developed, one dimensional flow through a straight duct is assumed. Equations for the isentropic expansion and for the adiabatic and the isothermal flow through a straight duct are given in textbooks on gas dynamics [1]. Denoting the stagnation conditions with zero (0), the state of the fluid in front of the membrane as 1, e.g., P_1 or Ma_1, and the state in the exit plane as 2, the ratio of P_1 to the stagnation pressure is given by the isentropic condition,

$$\frac{P_0}{P_1} = \left(1 + \frac{\gamma - 1}{2} Ma_1^2\right)^{\frac{\gamma}{\gamma - 1}}. \quad \text{(A)}$$

The mass flux is given by

$$\rho_1 v_1 = \sqrt{\frac{2\gamma}{\gamma - 1}\left(1 - \left(\frac{P_1}{P_0}\right)^{\frac{\gamma - 1}{\gamma}}\right)}\left(\frac{P_1}{P_0}\right)^{\frac{1}{\gamma}} \frac{P_0}{\sqrt{RT_0/M}} \quad \text{(B)}$$

and the Reynolds number is

$$Re = \frac{\rho_1 v_1 d}{\eta}. \quad \text{(C)}$$

For adiabatic flow through a long duct, the maximum length L_{max}, i.e., the length for which in the exit plane Mach number unity is reached, $Ma_2 = 1$, is given by [1, eq. (6.21)]

$$\frac{L_{max} f_D}{d} = \frac{1 - Ma^2}{\gamma Ma^2} + \frac{\gamma + 1}{2\gamma} ln\left(\frac{(\gamma + 1)Ma^2}{2\left(1 + (\gamma - 1)Ma^2/2\right)}\right), \quad \text{(D)}$$

where L_{max} counts from the position where the Ma is evaluated, and f_D is the Darcy-Weissenbach friction factor. Here, the flow is always laminar, hence $f_D = 64/Re$.

To compute the adiabatic, compressible flow through the pores, first the critical downstream pressure P*, i.e., the pressure at which Ma = 1 in the exit plane is reached, is determined. Setting f_D to 64/Re in eq. (D), substituting for Re from eq. (C) and further substituting for $\rho_1 v_1$ and P_1 from eqs. (B) and (A), respectively, yields a function $L_{max}/d = f(Ma_1, P_0)$. Finding the root of the equation $L/d - L_{max}/d = 0$ yields Ma_1, the Mach number in the entry plane of the pore, for which $P_2 = P^*$ is reached, i.e., $Ma_2 = 1$. The remaining values are determined from substituting back for Ma_1 into eqs. (A) to (C).

The pressure in the exit plane is determined from an expression for the ratio of the pressure at an arbitrary location in the duct to the critical pressure 1, eq. (6.22),

$$\frac{P}{P^*} = \frac{1}{Ma}\sqrt{\frac{\gamma + 1}{2\left(1 + (\gamma - 1)Ma^2/2\right)}}. \quad \text{(E)}$$

From above, Ma_1 and P_1 is known, hence eq. (E) yields P*.

If the downstream pressure P_e is smaller than the critical pressure P*, this is already the solution. The flow is choked, $Ma_2 = 1$ is reached in the exit plane of the pore, and the gas expands to the downstream pressure after leaving the pore.

If the downstream pressure P_e is larger than P*, the flow is calculated by combining eqs. (A) and (E),

$$\frac{P_0}{P_2} = \frac{P_0 P_1 P^*}{P_1 P^* P_2} = \frac{Ma_2}{Ma_1}\sqrt{\left(1 + \frac{\gamma - 1}{2}Ma_1^2\right)\left(1 + \frac{\gamma - 1}{2}Ma_2^2\right)}. \quad \text{(F)}$$

With P_0 and P_2 known, eq. (F) is an quadratic in either Ma_1 or Ma_2. Likewise,

$$L = L_{max}(Ma_1) - L_{max}(Ma_2) \quad \text{(G)}$$

is, after substitution from eqs. (A) to (C), also an equation in the two unknowns Ma_1 and Ma_2. Eqs. (F) and (G) can be solved to yield the Mach numbers, P_1 from eq. (A) and the mass flux from eq. (B).

For isothermal flow, eqs. (D) and (E) must be replaced by [1, eq. (6.36)]

$$\frac{L_{max} f_D}{d} = \frac{1}{\gamma Ma^2} - 1 + ln\left(\gamma Ma^2\right) \quad \text{(H)}$$

and [1, eq. (6.39)]

$$\frac{P}{P^*} = \frac{1}{\sqrt{\gamma} Ma}, \quad \text{(I)}$$

respectively, noting that the maximum Mach number for isothermal flow is $1/\sqrt{\gamma}$. Otherwise, the solution of the equations for isothermal flow is obtained analogously.

For comparison, the results for isentropic flow throughout are obtained by applying eq. (A) to the downstream state,

$$\frac{P_0}{P_2} = \left(1 + \frac{\gamma - 1}{2}Ma_2^2\right)^{\frac{\gamma}{\gamma - 1}} \quad \text{(J)}$$

and

$$\frac{P_0}{P^*} = \left(1 + \frac{\gamma - 1}{2}\right)^{\frac{\gamma}{\gamma - 1}}. \quad \text{(K)}$$

Figure SI4 shows the ratio of the critical pressure to the background pressure, P^*/P_e versus the Knudsen number at the mean pressure in the pores. Figure SI 4 shows the results for adiabatic flow, eqs. (A) to (G), isothermal flow, eqs. (A) to (C), (H), (I), and isentropic flow, eqs. (J) and

(K). For P^*/P_e larger than 1, the flow is choked, and the velocity that is computed assuming a combination of viscous and Knudsen flow might be in error. However, for Knudsen numbers approaching and exceeding unity, a viscous, continuum description becomes more and more inaccurate.

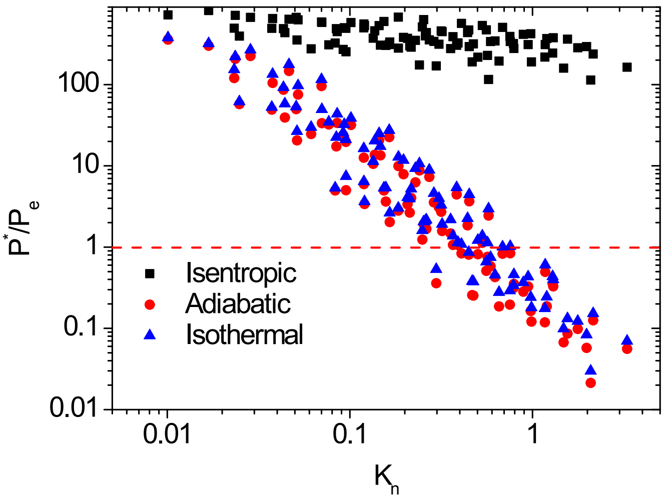

Figure SI 4. The dependence of P^*/P_e ratio vs. average Knudsen number for isentropic, adiabatic, isothermal flow.

References

1: A. H. Shapiro, The Dynamics and Thermodynamics of Compressible Fluid Flow, Ronald Press, 1953
